# Crystal structure and Hirshfeld surface analysis of 4′-chloro-5-oxo-*N*-phenyl-3-(thio­phen-2-yl)-2,3,4,5-tetra­hydro-[1,1′-biphen­yl]-4-carboxamide monohydrate

**DOI:** 10.1107/S2056989026007826

**Published:** 2026-08-04

**Authors:** Farid N. Naghiyev, Victor N. Khrustalev, Elena Yu. Nevskaya, Mehmet Akkurt, Jamshid M. Ashurov, Hamouda Adam Hamouda, İbrahim G. Mamedov

**Affiliations:** a Faculty of Chemistry, Baku State University, Z. Khalilov str. 23, Az, 1148, Baku, Azerbaijan; b Peoples’ Friendship University of Russia (RUDN University) Miklukho-Maklay St 6 Moscow 117198 Russian Federation; c N. D. Zelinsky Institute of Organic Chemistry RAS, Leninsky Prosp. 47, Moscow, 119991, Russian Federation; d Department of Physics, Faculty of Sciences, Erciyes University, 38039 Kayseri, Türkiye; e Institute of Bioorganic Chemistry, Academy of Sciences of Uzbekistan, M. Ulugbek Str.,83, Tashkent, 100125, Uzbekistan; f Department of Chemistry, Faculty of Science, University of Kordofan, Sudan; Institute of Chemistry, Chinese Academy of Sciences

**Keywords:** crystal structure, face-to-face π–π inter­actions, thio­phene ring, disorder, Hirshfeld surface analysis

## Abstract

The title mol­ecule, C_23_H_18_ClNO_2_S·H_2_O, has a stable conformation with *S*(5) and *S*(6) rings formed by intra­molecular C—H⋯S and C—H⋯O hydrogen bonds. The water mol­ecule and the carboxyl oxygen atom of the main mol­ecule also make an O—H⋯O hydrogen bond. In the crystal, N—H⋯O, O—H⋯Cl, O—H⋯O and C—H⋯O hydrogen bonds connect mol­ecules to form a three-dimensional network. The mol­ecules are further connected by C—H⋯π inter­actions and face-to-face π–π inter­actions into layers parallel to the (001) plane.

## Abstract 2

In the crystal, inter­molecular N—H⋯O, O—H⋯Cl, O—H⋯O and C—H⋯O hydrogen bonds connect mol­ecules to form a three-dimensional network. The mol­ecules are further connected by C—H⋯π inter­actions and face-to-face π–π inter­actions into layers parallel to the (001) plane.

## 1. Chemical context

Michael addition and aldol condensation reactions play a significant role in carbon–carbon bond formation, providing highly efficient and versatile strategies for the construction of complex mol­ecular frameworks (Naghiyev *et al.*, 2023 ▸; Mamedov & Khalilov, 2025 ▸). These transformations enable the formation of new C—C bonds under relatively mild conditions, often with excellent regio- and stereoselectivity (Safarova *et al.*, 2023 ▸, 2024 ▸; Tüzün *et al.*, 2025 ▸). Owing to these advantages, Michael addition and aldol condensation reactions are widely employed in organic synthesis, particularly in the preparation of pharmaceuticals, natural products, and advanced functional materials (Naghiyev *et al.*, 2022 ▸, 2024 ▸; Karimli *et al.*, 2023 ▸). Among the products accessible through this synthetic approach, highly substituted cyclo­hexenone derivatives have attracted considerable attention owing to their structural diversity, widespread occurrence in both natural and synthetic compounds, and their utility as versatile inter­mediates in organic synthesis (Asgarova *et al.*, 2019 ▸; Abdel-Galil *et al.*, 2022 ▸). In addition to their synthetic importance, selected members of this class have been reported to exhibit a variety of biological activities, making cyclo­hexenone-based frameworks attractive targets in medicinal chemistry (Khan *et al.*, 2021 ▸; Shikhaliyev *et al.*, 2025 ▸). In particular, aryl-substituted cyclo­hexenone carboxamides represent valuable mol­ecular scaffolds for the synthesis of structurally diverse compounds and continue to attract inter­est in synthetic and structural chemistry. The incorporation of aromatic, heteroaromatic, and amide functionalities into the cyclo­hexenone framework further expands the scope for structural diversification and detailed investigations of their mol­ecular and crystal structures (Lakhrissi *et al.*, 2022 ▸; Nosova *et al.*, 2023 ▸). In this context, herein we report the crystal structure and Hirshfeld surface analysis of the title compound, C_23_H_18_ClNO_2_S·H_2_O (**4**), as part of our ongoing investigations (Askerov *et al.*, 2020 ▸; Naghiyev *et al.*, 2021 ▸; Khalilov *et al.*, 2011 ▸).

**Figure 1:**
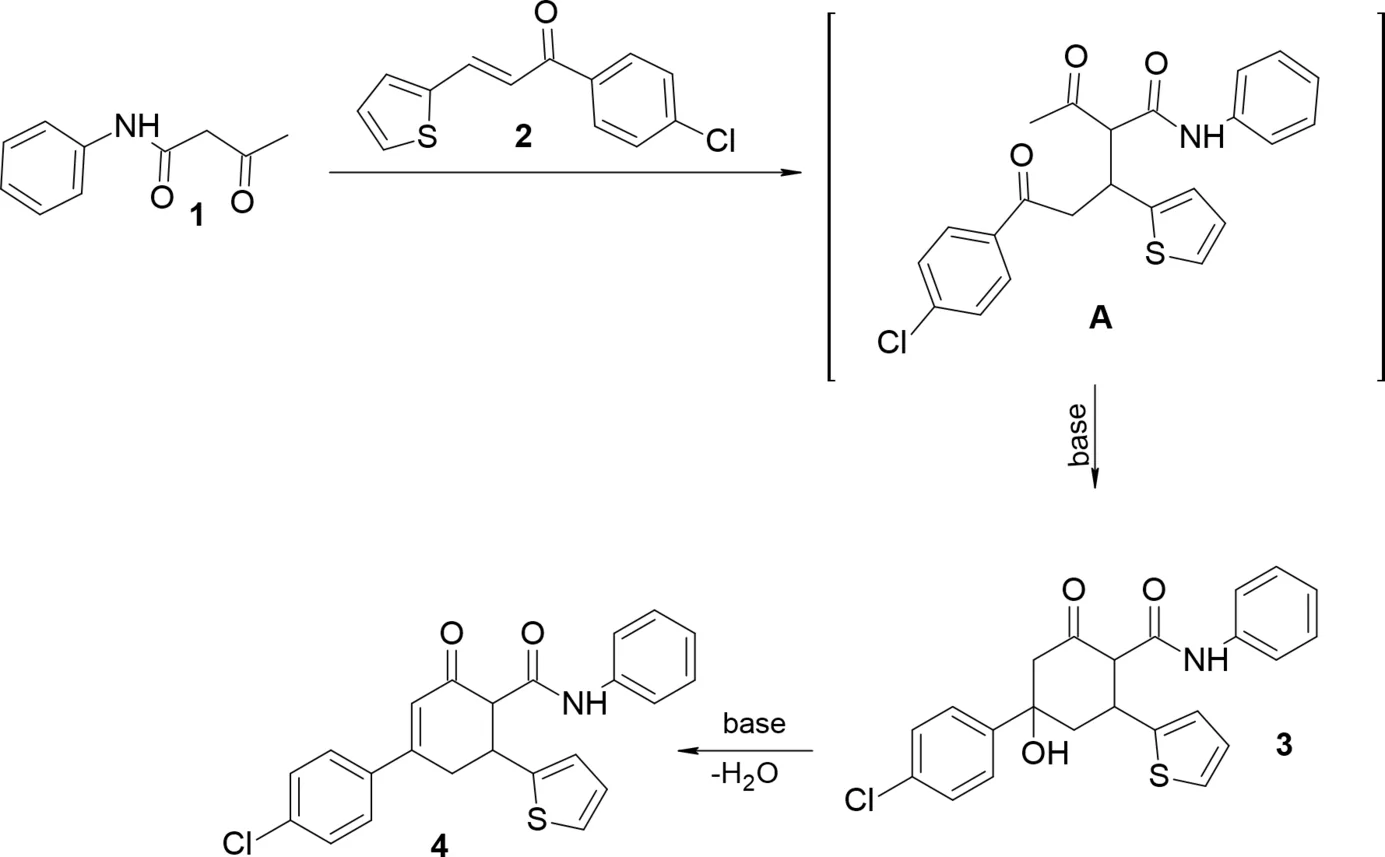
A reaction route for the formation of the title compound **4**.

A reaction route for the formation of the title compound **4** is depicted in Fig. 1 ▸. The reaction is initiated by the Michael addition of the acetoacetamide **1** to the α,β-unsaturated chalcone **2**, leading to the formation of inter­mediate **A**. Under basic conditions, inter­mediate **A** undergoes intra­molecular alkyl­ation to form compound **3**, which then undergoes an intra­molecular aldol condensation, resulting in the formation of the cyclo­hexane ring. Subsequent dehydration of this inter­mediate leads the corresponding cyclo­hexenone derivative **4**.
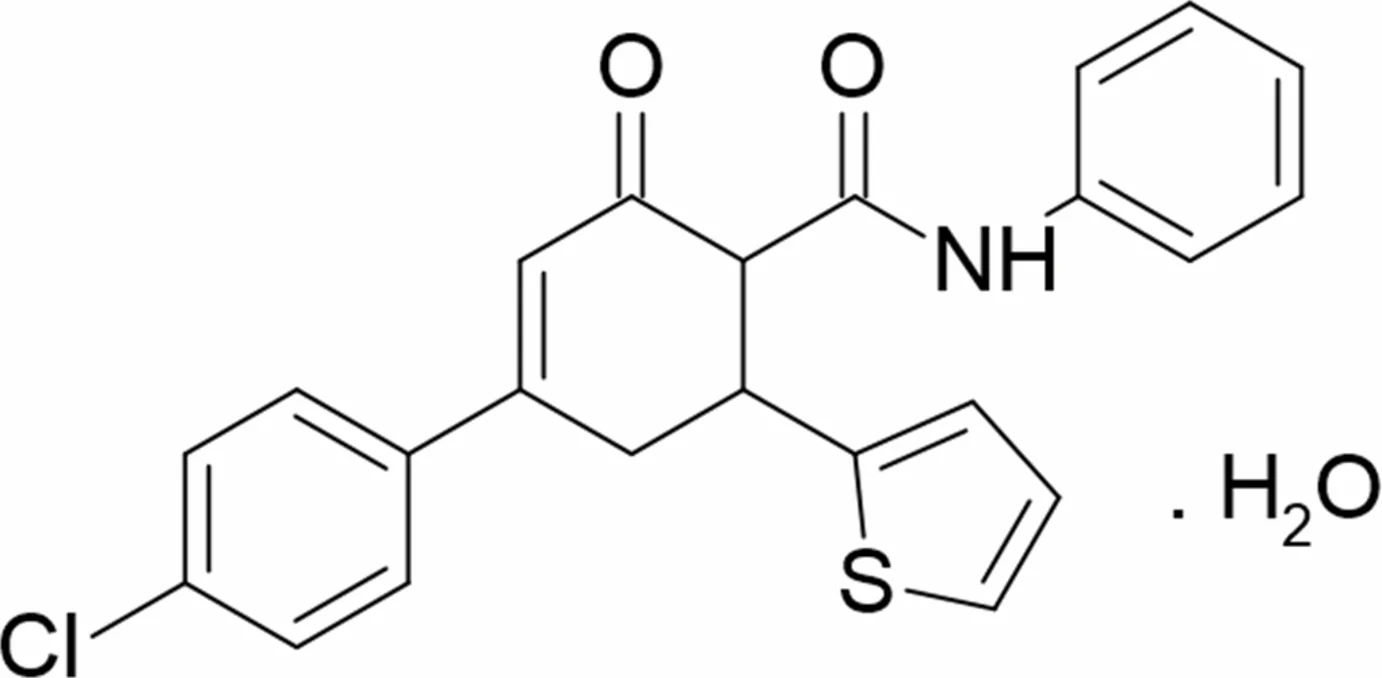


## 2. Structural commentary

**Figure 2:**
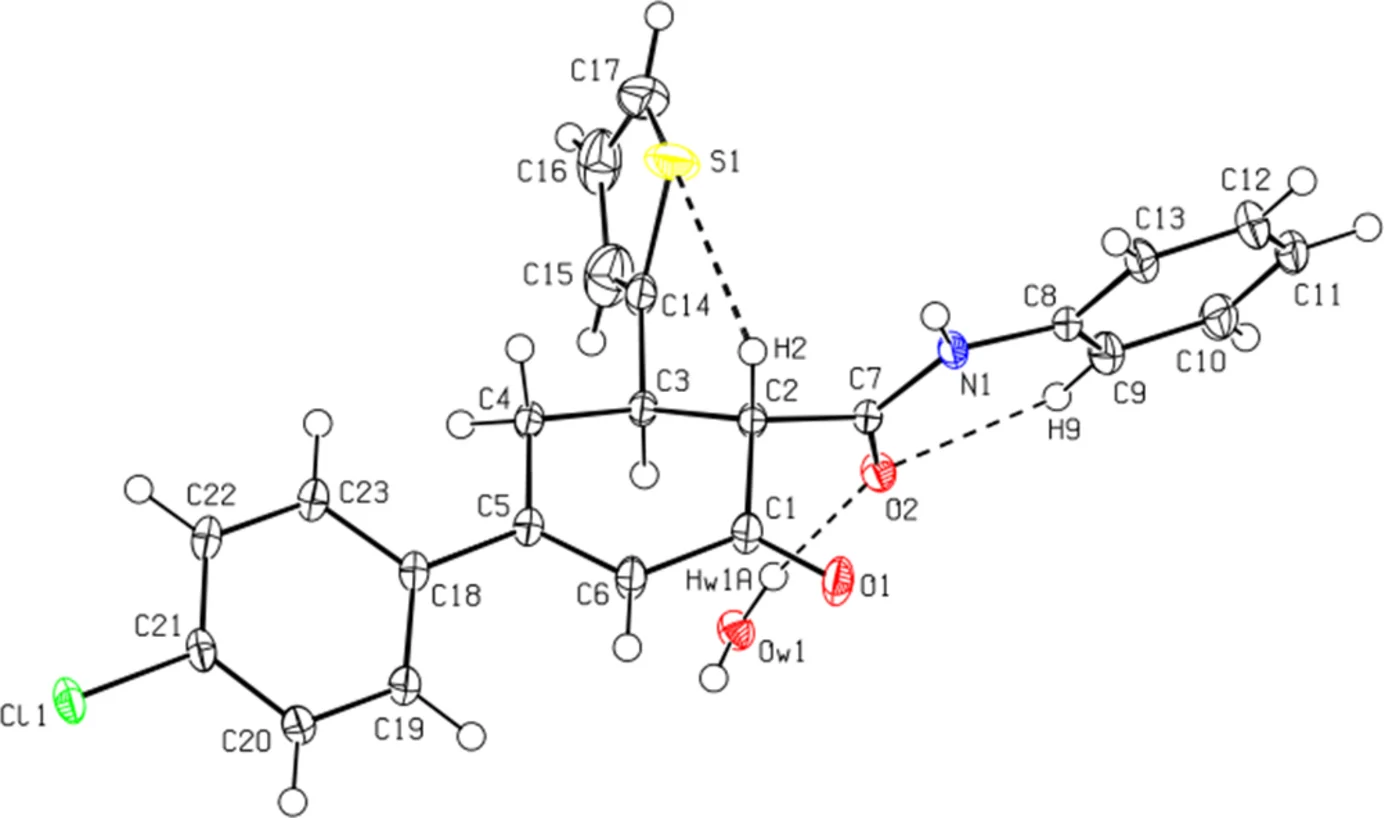
Mol­ecular structure of the title compound, with atom labelling. Displacement ellipsoids are drawn at the 50% probability level. For clarity, only one component of the disordered thio­phene ring is shown.

The conformation of the title mol­ecule is stable with the C—H⋯S and C—H⋯O intra­molecular hydrogen bonding, forming *S*(5) and *S*(6) rings (Fig. 2 ▸, Table 1 ▸; Bernstein *et al.*, 1995 ▸). Additionally, an O*w*1—H*w*1*A*⋯O2 hydrogen bond exists between the water mol­ecule and the main mol­ecule’s carboxyl oxygen atom. Each mol­ecule contains two stereogenic (chiral) centres: in the arbitrarily chosen asymmetric unit, C2 and C3 have *S* and *R* configurations, respectively. The thiophene ring (S1/C13–C17) is disordered by a 180° rotation over two orientations around the C3—C14 bond in a 0.5:0.5 ratio. The cyclo­hexene ring (C1–C6) is puckered with Cremer & Pople (1975 ▸) puckering parameters *Q*_T_ = 0.4622 (9) Å, θ = 54.10 (11)° and φ = 112.74 (14)°. The r.m.s plane (r.m.s. deviation = 0.005 Å) of the cyclo­hexane ring (C1–C6) subtends dihedral angles of 73.1 (2), 70.72 (5) and 25.34 (4) °, respectively, with the thio­phene (S1/C14–C17), phenyl (C8–C13) and chloro­phenyl (C18–C23) rings. The thio­phene ring subtends angles of 69.5 (2) and 86.6 (2)° with the phenyl and chloro­phenyl rings, respectively. The angle between the phenyl and chloro­phenyl rings is 84.58 (5)°. The bond lengths and angles in the title compound are in good agreement with those in the compounds discussed in the *Database survey* section.

## 3. Supra­molecular features

**Figure 3:**
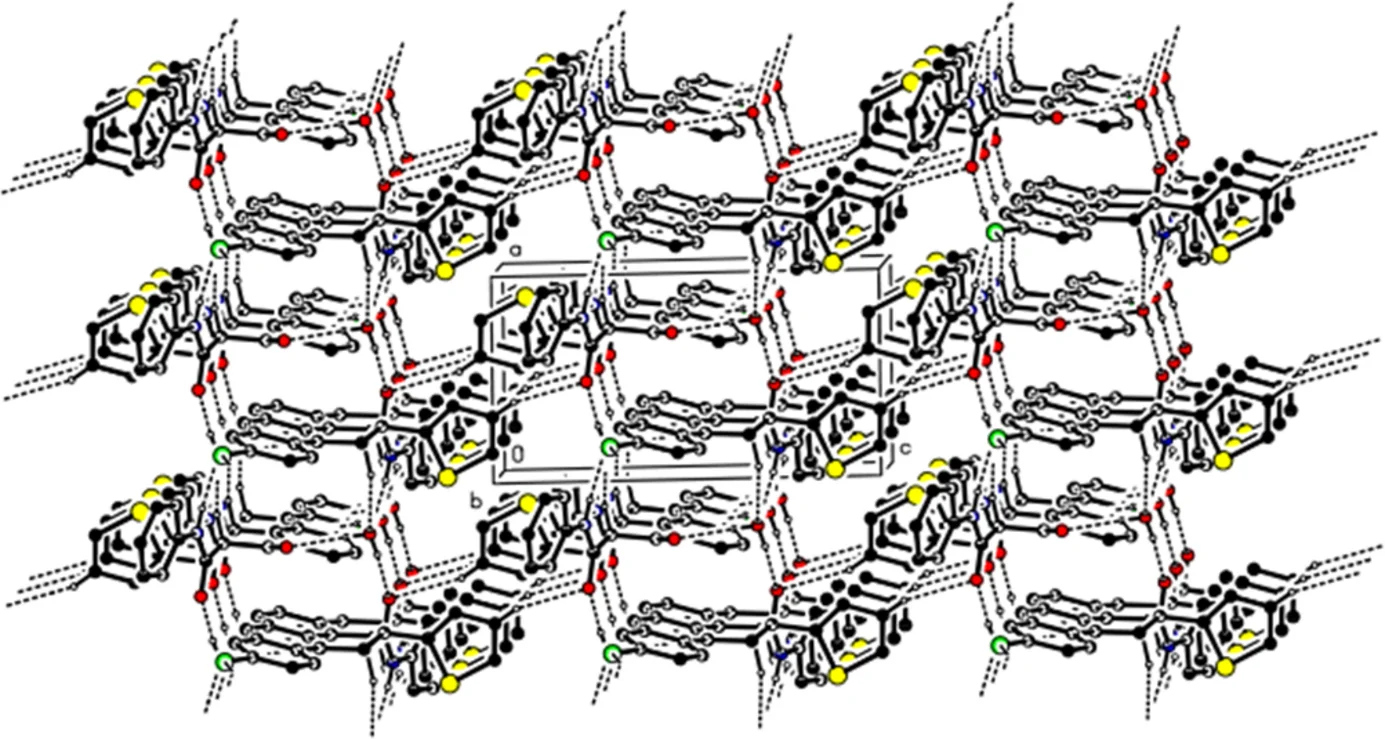
A view of the packing along the *b*-axis direction of the title compound with N—H⋯O, O—H⋯O, O—H⋯Cl and C—H⋯O hydrogen bonds shown as dashed lines. Hydrogen atoms that do not participate in hydrogen bonding are not displayed for clarity.

**Figure 5:**
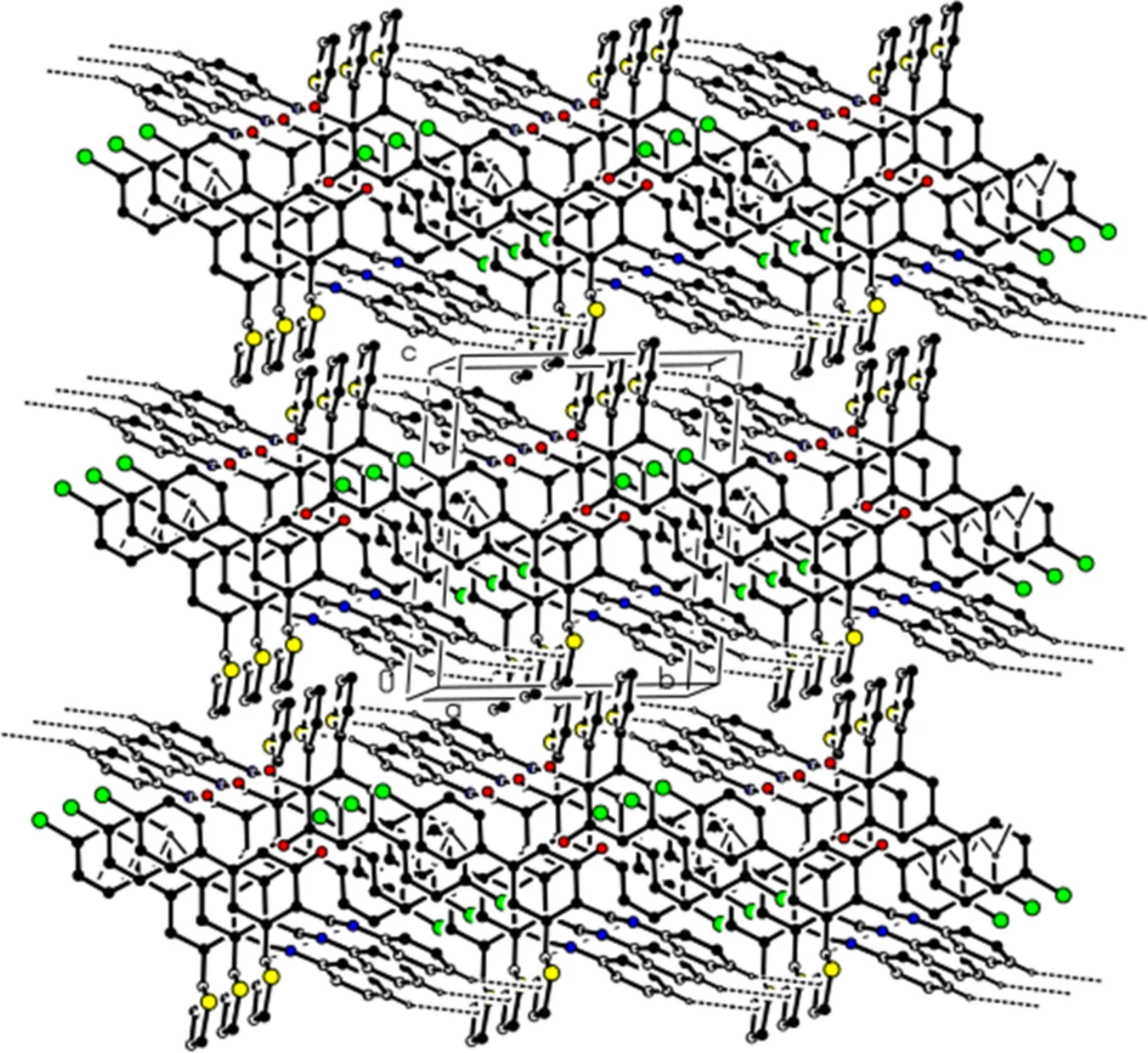
A view of the packing along the *a*-axis direction of the title compound with C—H⋯π and π–π inter­actions shown as dashed lines. Hydrogen atoms that do not engage in hydrogen bonding and water mol­ecules are not shown for clarity.

In the crystal, mol­ecules are linked to form a three-dimensional network *via* inter­molecular N1—H1⋯O*w*1, O*w*1—H*w*1*B*⋯Cl1, O*w*1—H*w*1*B*⋯O1, C2—H2⋯O*w*1, C16—H16⋯O2 and C17′—H17′⋯O2 hydrogen bonds (Figs. 3 ▸ and 4 ▸, Table 1 ▸). In addition, C—H⋯π inter­actions and face-to-face π–π inter­actions [*Cg*5⋯*Cg*5(−*x*, 2 − *y*, 1 − *z*) = 3.9020 (6) Å, slippage = 1.970 Å and *Cg*5⋯*Cg*5(1 − *x*, 2 − *y*, 1 − *z*) = 3.5513 (6) Å, slippage = 0.158 Å; where *Cg*5 is the centroid of the C18–C23 ring of the chloro­phenyl group] connect the mol­ecules, forming layers parallel to the (001) plane (Figs. 5 ▸ and 6 ▸, Table 1 ▸).

## 4. Hirshfeld surface analysis

**Figure 7:**
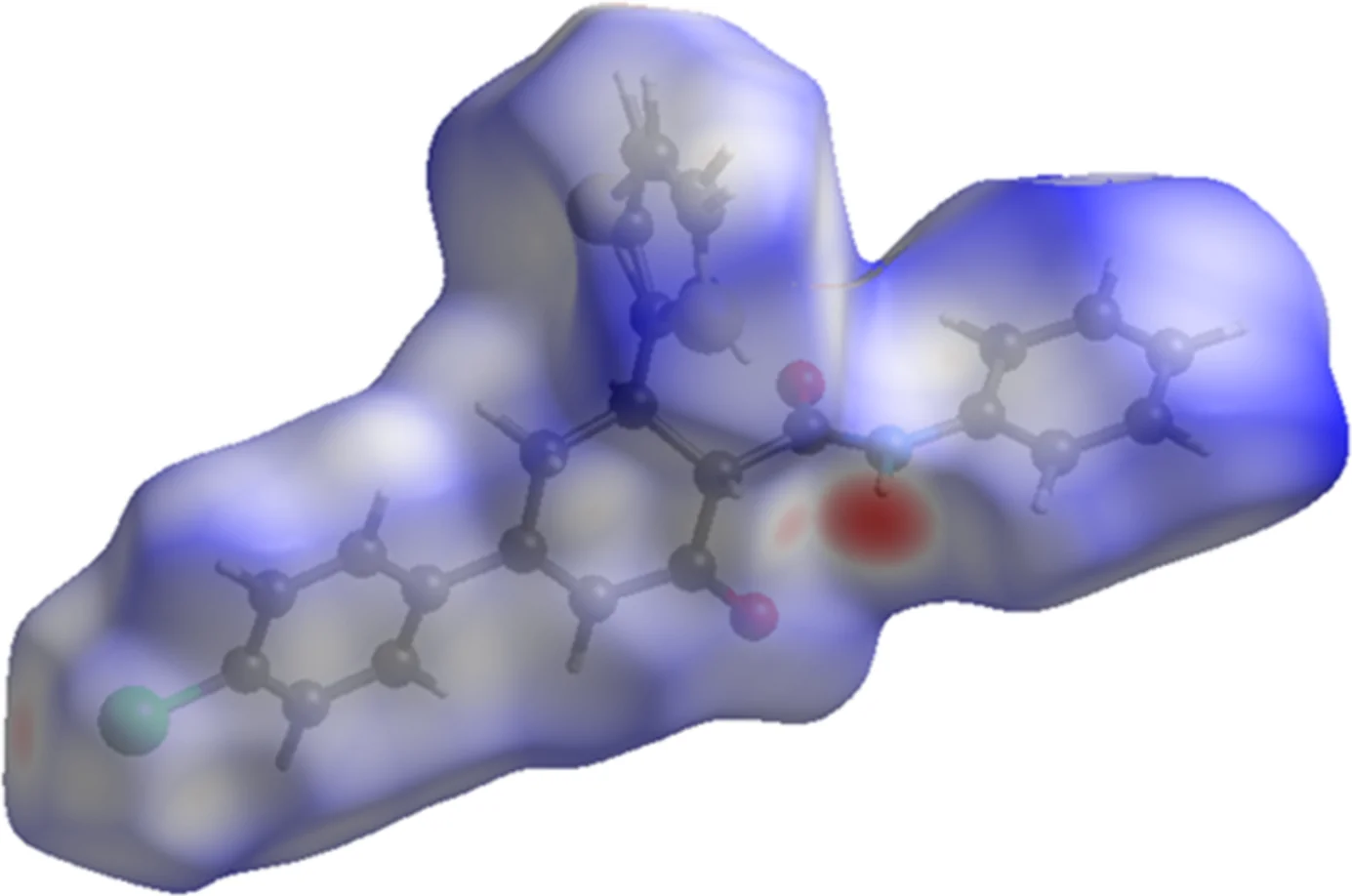
View of the three-dimensional Hirshfeld surface of the title compound plotted over *d*_norm_ in the range −0.6301 to +1.6037 a.u.

The Hirshfeld surfaces and two-dimensional fingerprint plots were produced using *Crystal Explorer 17.5* (Spackman *et al.*, 2021 ▸). Hirshfeld surfaces enable the presentation of inter­molecular inter­actions by indicating short and long contacts, as well as the relative strength of the inter­actions, with different colors and intensities. Fig. 7 ▸ displays the title compound’s three-dimensional Hirshfeld surface plotted over *d*_norm_ in the range −0.6301 to +1.6037 a.u. The red patch surrounding O1, O2, O*w*1 and Cl1 are caused by the N—H⋯O, O—H⋯Cl, O—H⋯O and C—H⋯O inter­actions (Table 1 ▸), which are crucial to the mol­ecular packing of the title mol­ecule.

**Figure 8:**
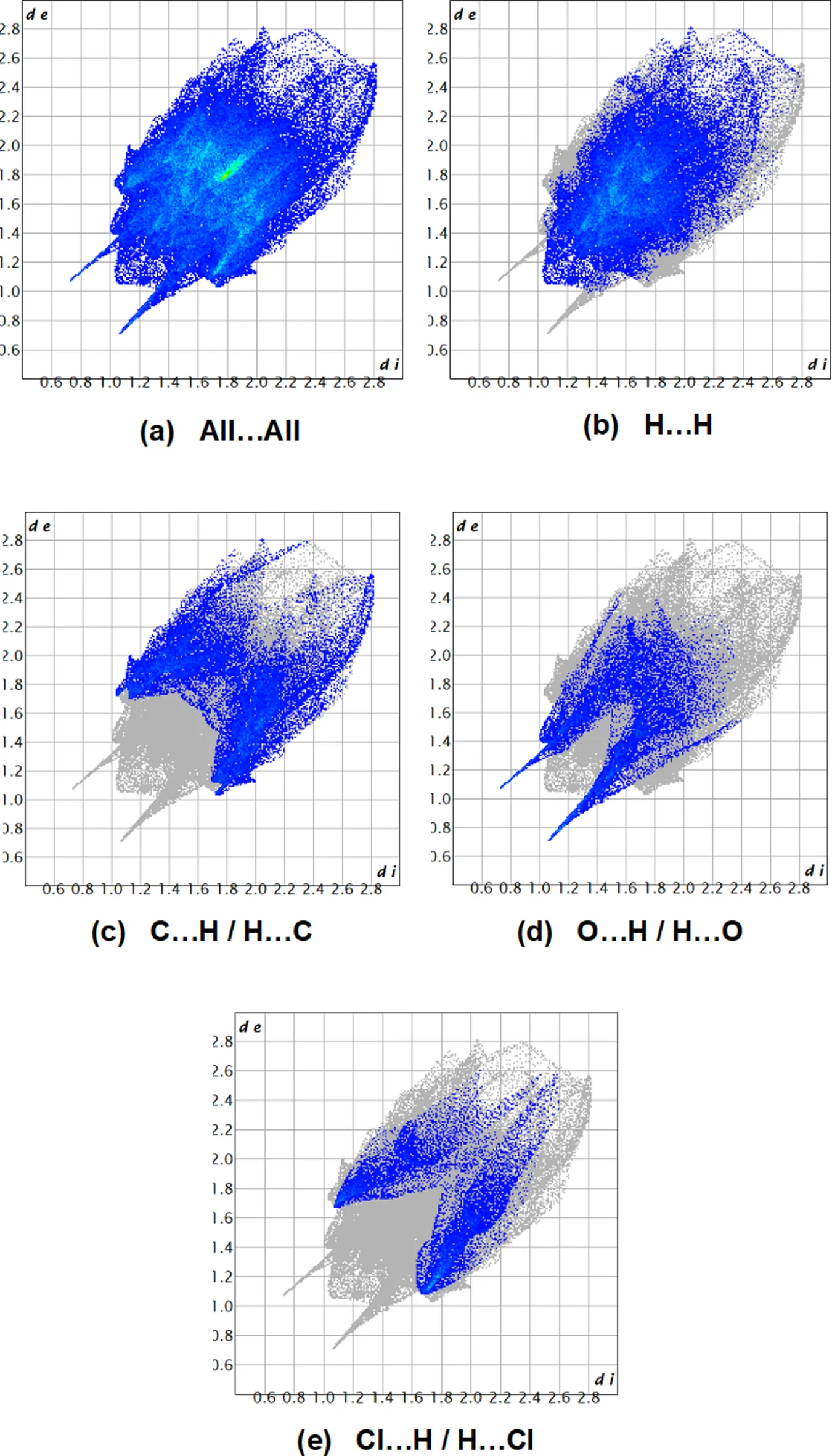
The full two-dimensional fingerprint plots for the title compound, showing (*a*) all inter­actions, and delineated into (*b*) H⋯H, (*c*) C⋯H/H⋯C, (*d*) O⋯H/H⋯O and (*e*) Cl⋯H/H⋯Cl inter­actions. The *d*_i_ and *d*_e_ values are the closest inter­nal and external distances (in Å) from given points on the Hirshfeld surface.

Fig. 8 ▸*a* shows the overall two-dimensional fingerprint plot for the title compound, while Fig. 8 ▸*b*–*e* show those that are divided into H⋯H, C⋯H/H⋯C, O⋯H/H⋯O and Cl⋯H/H⋯Cl contacts. Tables 1 ▸ and 2 ▸ provide numerical information of the various contacts. The percentage contributions to the Hirshfeld surfaces from the different inter­atomic contacts are as follows: H⋯H (Fig. 8 ▸*b*; 43.2%), C⋯H/H⋯C (Fig. 8 ▸*c*; 22.2%), O⋯H/H⋯O (Fig. 8 ▸*d*; 12.5%) and Cl⋯H/H⋯Cl (Fig. 8 ▸*e*; 9.8%). The C⋯C (4.8%), S⋯H/H⋯S (3.2%), O⋯C/C⋯O (1.3%), N⋯H/H⋯N (1.2%), Cl⋯O/O⋯Cl (0.8%), Cl⋯C/C⋯Cl (0.7%) and O⋯O (0.3%) contacts are additional modest contributions to the Hirshfeld surface.

## 5. Database survey

A search of the Cambridge Structural Database (CSD, version 6.00, updated April 2025; Groom *et al.*, 2016 ▸) for the title compound revealed two compounds: 5-oxo-*N*-phenyl-3-(thio­phen-2-yl)-2,3,4,5-tetra­hydro-1,1′-biphenyl-4-carboxamide (CSD ref code AHEMIN: Aliyeva *et al.*, 2025 ▸) and dimethyl-4′-bromo-3-oxo-5-(thio­phen-2-yl)-3,4,5,6-tetra­hydro­[1,1′-biphen­yl]- 2,4-di­carboxyl­ate (WOMWUU: Naghiyev *et al.*, 2024 ▸). AHEMIN and WOMWUU crystallize in the monoclinic *Cc* and *P*2/_1_*c* space groups, respectively.

The two mol­ecules in the asymmetric unit of AHEMIN have different carboxamide moiety conformations. Inter­molecular N—H⋯O hydrogen bonds in the crystal connect the mol­ecules into chains that propagate parallel to the *c*-axis. While there are no π–π inter­actions between the mol­ecules, there are weak C—H⋯π(ring) inter­actions. In WOMWUU, mol­ecules form ribbons in the *b*-axis direction as a result of inter­molecular C—H⋯S hydrogen bonds with 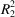
(10) ring motifs. van der Waals forces between the ribbons maintain the cohesiveness of the crystal structure, whereas C—H⋯π inter­actions consolidate the ribbon structure. The thio­phene group is disordered by a 180° rotation around one bond.

## 6. Synthesis and crystallization

A solution of 0.443 g (0.0025 mol) acetoacetanilide and 0.623 g (0.0025 mol) 1-phenyl-3-(thio­phen-2-yl)prop-2-en-1-one (5.10 mmol) in 30 ml ethanol (10 ml) was stirred for 1 h. After that, 3 drops of methyl­piperazine were added to the solution and the mixture was then stirred for 3 h while the course of reaction was monitored by TLC. After the reaction completion, the solvent was removed under reduced pressure and the residue was recrystallized from an ethanol–water mixture (m.p. = 391 K, yield 73%).

^1^H NMR (300 MHz, acetone-*d*_6_, δ, ppm.): 2.22 and 2.72 (*d-d*, 2H, CH_2_, ^2^*J*_H–H_ = 16.2 Hz, ^3^*J*_H–H_ = 8.3 Hz), 3.54 (*t-t*, 1H, CH, ^3^*J*_H–H_ = 8.3 Hz, ^3^*J*_H–H_ = 10.3 Hz), 3.78.15 (*d*, 1H, CH, ^3^*J*_H–H_ = 10.3 Hz), 6.59–7.67 (*m*, 13H, arom. and =CH), 9.56 (*s*, 1H, NH); ^13^C NMR (75 MHz, acetone-*d*_6_, δ, ppm.): 31.26 (CH), 44.45 (CH_2_), 52.87 (CH), 119.08 (CH_arom._), 119.20 (CH_arom._), 120.35 (C_arom._), 123.17 (=CH), 127.00 (CH_arom._), 128.69 (CH_arom._), 129.47 (CH_arom._), 130.97 (CH_arom._), 132.13 (C_arom._), 131.13 (CH_arom._), 133.43 (C_arom._), 131.90 (CH_arom._), 138.36 (C_arom._), 147.33 (C_quat.._), 167.27 (CO), 194.27 (CO).

## 7. Refinement

Crystal data, data collection and structure refinement details are summarized in Table 3 ▸. All C-bound hydrogen atoms were positioned geometrically (C—H = 0.95–1.00 Å) and refined using a riding model, with *U*_iso_(H) = 1.2*U*_eq_(C). The H atoms of the water mol­ecule and NH group were located from difference-Fourier maps and refined isotopically with dependent isotropic thermal parameters with *U*_iso_(H) = 1.2*U*_eq_(N) and *U*_iso_(H) = 1.5*U*_eq_(O). The thio­phene ring (S1/C13–C17) is disordered by a 180° rotation over two orientations around the C3—C14 bond in a 0.5:0.5 ratio. Using the FLAT command, both components of the disordered thio­phene ring were restricted to the same plane. In addition, the DFIX command was used to restrain the S—C distances S1—C17, S1—C14, S1′—C17′ and S1′—C14 to fix their bond lengths to 1.72 Å. Furthermore, DELU, SIMU and ISOR commands were used in the refinement to restrain the thermal factors of the disordered C15 S1′, C16 C17′, C17 C16′ and C15′ S1components. Using the SADI command, the C—C lengths of the thio­phene rings were pushed to be the same.

## Supplementary Material

Crystal structure: contains datablock(s) I. DOI: 10.1107/S2056989026007826/nx2038sup1.cif

Structure factors: contains datablock(s) I. DOI: 10.1107/S2056989026007826/nx2038Isup2.hkl

CCDC reference: 2577688

Additional supporting information:  crystallographic information; 3D view; checkCIF report

## Figures and Tables

**Figure 4:**
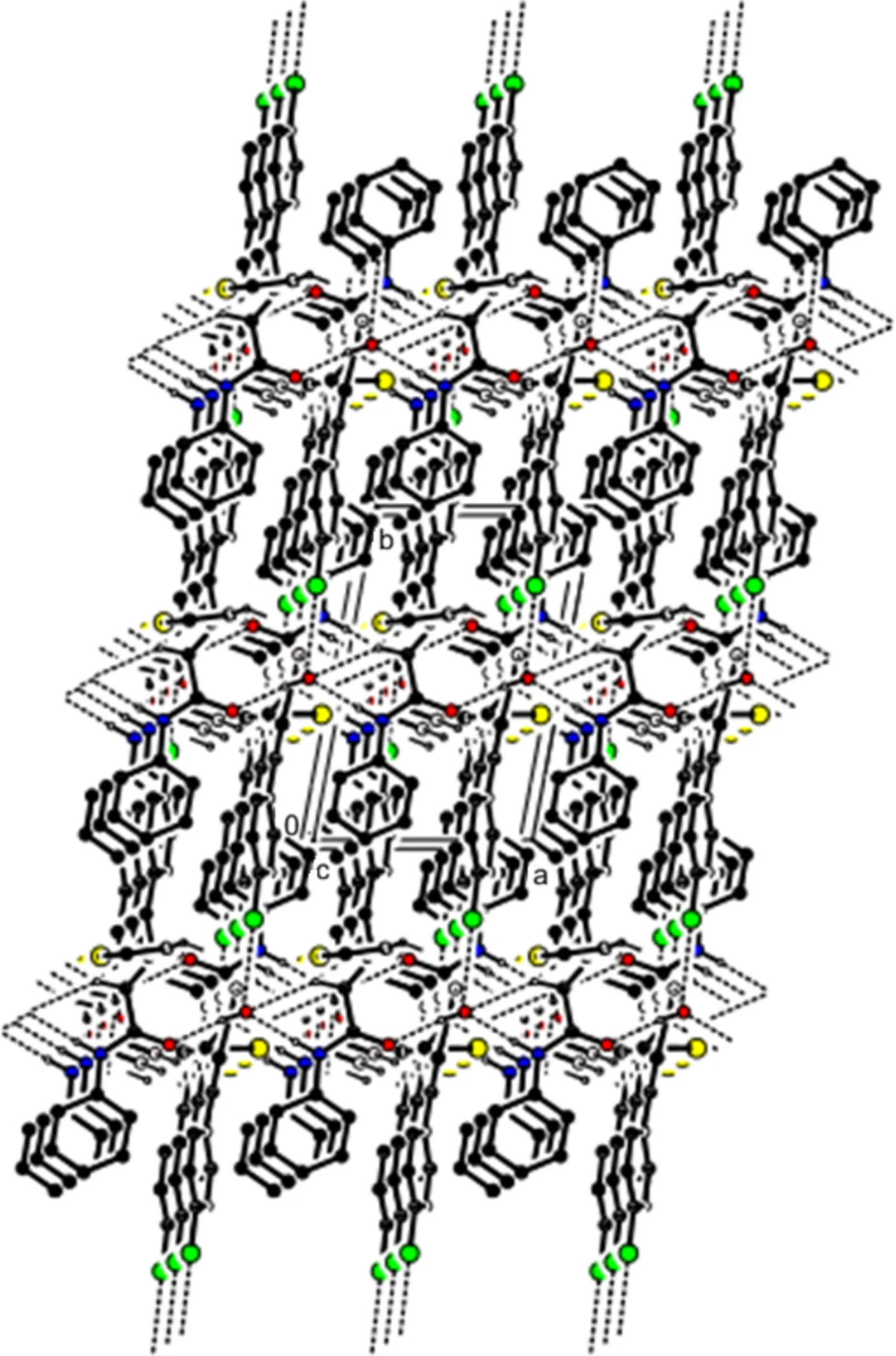
A view of the packing along the *c*-axis direction of the title compound with N—H⋯O, O—H⋯O, O—H⋯Cl and C—H⋯O hydrogen bonds shown as dashed lines.

**Figure 6:**
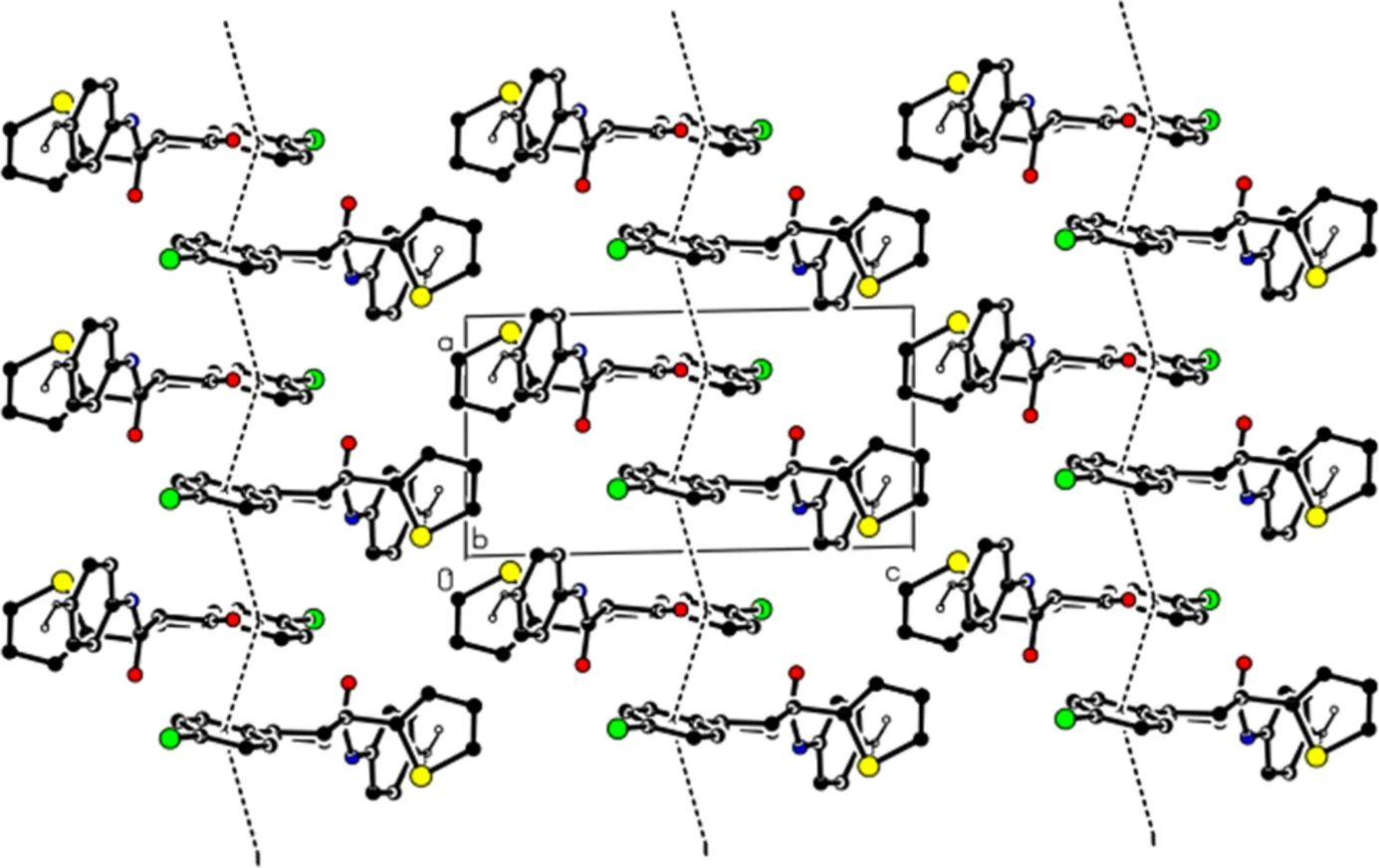
A view of the packing along the *b*-axis direction of the title compound with C—H⋯π and π–π inter­actions shown as dashed lines.

**Table 1:** Hydrogen-bond geometry (Å, °) *Cg*1 is the centroid of the major component of the disordered thio­phene ring (S1/C14–C17).

*D*—H⋯*A*	*D*—H	H⋯*A*	*D*⋯*A*	*D*—H⋯*A*
N1—H1⋯O*w*1^i^	0.881 (14)	1.953 (14)	2.8317 (10)	175.2 (12)
O*w*1—H*w*1*A*⋯O2	0.84 (2)	1.93 (2)	2.7681 (9)	171 (2)
O*w*1—H*w*1*B*⋯Cl1^ii^	0.83 (2)	2.81 (2)	3.0653 (7)	100 (1)
O*w*1—H*w*1*B*⋯O1^iii^	0.83 (2)	2.07 (2)	2.8908 (9)	172 (2)
C2—H2⋯S1	1.00	2.80	3.1912 (15)	104
C2—H2⋯O*w*1^i^	1.00	2.50	3.3269 (10)	139
C9—H9⋯O2	0.95	2.32	2.9124 (11)	120
C16—H16⋯O2^iv^	0.95	2.55	3.423 (6)	152
C17′—H17′⋯O2^iv^	0.95	2.58	3.511 (5)	166
C11—H11⋯*Cg*1^v^	0.95	2.91	3.801 (2)	158

Symmetry codes: (i) 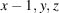; (ii) 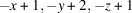; (iii) 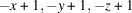; (iv) 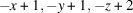; (v) 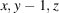.

**Table 2:** Summary of short inter­atomic contacts (Å)

Contact	Distance	Symmetry operation
H23⋯C12	2.99	*x*, 1 + *y*, *z*
Cl1⋯H*w*1*B*	2.81	1 − *x*, 2 − *y*, 1 − *z*
H3⋯Cl1	2.83	1 − *x*, 2 − *y*, 1 − *z*
H4*A*⋯Cl1	2.88	−*x*, 2 − *y*, 1 − *z*
*H16′⋯C8	2.89	−*x*, 1 − *y*, 2 − *z*
*H16⋯H9	2.48	1 − *x*, 1 − *y*, 2 − *z*
O1⋯H*w*1*B*	2.07	1 − *x*, 1 − *y*, 1 − *z*
O2⋯H*w*1*A*	1.93	*x*, *y*, *z*
H19⋯O2	2.75	1 − *x*, 1 − *y*, 1 − *z*
H1⋯O*w*1	1.95	−1 + *x*, *y*, *z*

The prefix * indicates a disordered atom.

**Table 3:** Experimental details

Crystal data	
Chemical formula	C_23_H_20_ClNO_3_S
*M*_r_	425.91
Crystal system, space group	Triclinic, *P* 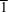
Temperature (K)	100
*a*, *b*, *c* (Å)	7.1577 (3), 11.1279 (4), 13.2067 (5)
α, β, γ (°)	85.412 (1), 87.973 (1), 79.348 (1)
*V* (Å^3^)	1030.25 (7)
*Z*	2
Radiation type	Mo *K*α
μ (mm^−1^)	0.31
Crystal size (mm)	0.24 × 0.22 × 0.13

Data collection	
Diffractometer	Bruker D8 QUEST PHOTON-III area detector
Absorption correction	Multi-scan (*SADABS*; Krause *et al.*, 2015 ▸).
*T*_min_, *T*_max_	0.707, 0.746
No. of measured, independent and observed [*I* > 2σ(*I*)] reflections	29949, 7470, 6538
*R*_int_	0.026
(sin θ/λ)_max_ (Å^−1^)	0.758

Refinement	
*R*[*F*^2^ > 2σ(*F*^2^)], *wR*(*F*^2^), *S*	0.033, 0.096, 1.04
No. of reflections	7470
No. of parameters	307
No. of restraints	171
H-atom treatment	H atoms treated by a mixture of independent and constrained refinement
Δρ_max_, Δρ_min_ (e Å^−3^)	0.47, −0.32

Computer programs: *APEX3* and *SAINT* (Bruker, 2018 ▸), *SHELXT* (Sheldrick, 2015*a* ▸), *SHELXL* (Sheldrick, 2015*b* ▸), *ORTEP-3 for Windows* (Farrugia, 2012 ▸) and *PLATON* (Spek, 2020 ▸).

## supplementary crystallographic information

### 4'-chloro-5-oxo-N-phenyl-3-(thiophen-2-yl)-2,3,4,5-tetrahydro-[1,1'-biphenyl]-4-carboxamide monohydrate Crystal data

**Table:**

C_23_H_20_ClNO_3_S	*Z* = 2
*M**_r_* = 425.91	*F*(000) = 444
Triclinic, *P*1	*D*_x_ = 1.373 Mg m^−^^3^
*a* = 7.1577 (3) Å	Mo *K*α radiation, λ = 0.71073 Å
*b* = 11.1279 (4) Å	Cell parameters from 9950 reflections
*c* = 13.2067 (5) Å	θ = 2.3–32.6°
α = 85.412 (1)°	µ = 0.31 mm^−^^1^
β = 87.973 (1)°	*T* = 100 K
γ = 79.348 (1)°	Prism, colourless
*V* = 1030.25 (7) Å^3^	0.24 × 0.22 × 0.13 mm

### 4'-chloro-5-oxo-N-phenyl-3-(thiophen-2-yl)-2,3,4,5-tetrahydro-[1,1'-biphenyl]-4-carboxamide monohydrate Data collection

**Table:**

Bruker D8 QUEST PHOTON-III area detector diffractometer	6538 reflections with *I* > 2σ(*I*)
Radiation source: fine-focus sealed X-ray tube	*R*_int_ = 0.026
φ and ω scans	θ_max_ = 32.6°, θ_min_ = 2.3°
Absorption correction: multi-scan (SADABS; Krause *et al.*, 2015).	*h* = −10→10
*T*_min_ = 0.707, *T*_max_ = 0.746	*k* = −16→16
29949 measured reflections	*l* = −19→19
7470 independent reflections	

### 4'-chloro-5-oxo-N-phenyl-3-(thiophen-2-yl)-2,3,4,5-tetrahydro-[1,1'-biphenyl]-4-carboxamide monohydrate Refinement

**Table:**

Refinement on *F*^2^	Primary atom site location: difference Fourier map
Least-squares matrix: full	Secondary atom site location: difference Fourier map
*R*[*F*^2^ > 2σ(*F*^2^)] = 0.033	Hydrogen site location: mixed
*wR*(*F*^2^) = 0.096	H atoms treated by a mixture of independent and constrained refinement
*S* = 1.04	*w* = 1/[σ^2^(*F*_o_^2^) + (0.0492*P*)^2^ + 0.2767*P*] where *P* = (*F*_o_^2^ + 2*F*_c_^2^)/3
7470 reflections	(Δ/σ)_max_ = 0.001
307 parameters	Δρ_max_ = 0.47 e Å^−^^3^
171 restraints	Δρ_min_ = −0.32 e Å^−^^3^

### 4'-chloro-5-oxo-N-phenyl-3-(thiophen-2-yl)-2,3,4,5-tetrahydro-[1,1'-biphenyl]-4-carboxamide monohydrate Special details

**Table:**

Geometry. All esds (except the esd in the dihedral angle between two l.s. planes) are estimated using the full covariance matrix. The cell esds are taken into account individually in the estimation of esds in distances, angles and torsion angles; correlations between esds in cell parameters are only used when they are defined by crystal symmetry. An approximate (isotropic) treatment of cell esds is used for estimating esds involving l.s. planes.

### 4'-chloro-5-oxo-N-phenyl-3-(thiophen-2-yl)-2,3,4,5-tetrahydro-[1,1'-biphenyl]-4-carboxamide monohydrate Fractional atomic coordinates and isotropic or equivalent isotropic displacement parameters (Å^2^)

**Table:**

	*x*	*y*	*z*	*U*_iso_*/*U*_eq_	Occ. (<1)
C1	0.25524 (13)	0.54707 (8)	0.56920 (7)	0.01765 (15)	
C2	0.23078 (11)	0.53684 (7)	0.68443 (6)	0.01457 (14)	
H2	0.091669	0.556861	0.701114	0.017*	
C3	0.32834 (12)	0.62985 (7)	0.73273 (6)	0.01465 (14)	
H3	0.468240	0.607450	0.718973	0.018*	
C4	0.25951 (12)	0.75980 (7)	0.68385 (6)	0.01542 (14)	
H4A	0.126226	0.788477	0.705986	0.019*	
H4B	0.337635	0.815674	0.708601	0.019*	
C5	0.27020 (12)	0.76730 (7)	0.56990 (6)	0.01545 (14)	
C6	0.27143 (14)	0.66615 (8)	0.51952 (7)	0.02000 (16)	
H6	0.283576	0.673274	0.447529	0.024*	
C7	0.30438 (12)	0.40503 (7)	0.72605 (6)	0.01440 (14)	
C8	0.18480 (13)	0.21554 (7)	0.78940 (7)	0.01687 (15)	
C9	0.35133 (14)	0.14688 (8)	0.83063 (7)	0.02207 (17)	
H9	0.465042	0.179672	0.827866	0.026*	
C10	0.34922 (16)	0.02935 (9)	0.87605 (8)	0.02666 (19)	
H10	0.462878	−0.017716	0.903714	0.032*	
C11	0.18444 (17)	−0.02001 (9)	0.88158 (8)	0.0278 (2)	
H11	0.184360	−0.099511	0.913681	0.033*	
C12	0.02000 (17)	0.04840 (9)	0.83956 (9)	0.0293 (2)	
H12	−0.093377	0.015232	0.842451	0.035*	
C13	0.01940 (14)	0.16499 (9)	0.79328 (8)	0.02353 (18)	
H13	−0.093866	0.210660	0.764102	0.028*	
C14	0.29646 (15)	0.62858 (8)	0.84584 (7)	0.02117 (17)	
S1	0.07641 (17)	0.62692 (14)	0.90037 (9)	0.0457 (3)	0.5
C15	0.4188 (7)	0.6373 (4)	0.9170 (4)	0.0418 (9)	0.5
H15	0.548435	0.636045	0.898030	0.050*	0.5
C16	0.3628 (7)	0.6481 (6)	1.0190 (5)	0.0381 (10)	0.5
H16	0.433302	0.663471	1.074719	0.046*	0.5
C17	0.1850 (8)	0.6312 (7)	1.0167 (5)	0.0408 (12)	0.5
H17	0.115541	0.621279	1.078447	0.049*	0.5
S1'	0.46619 (13)	0.65996 (9)	0.92283 (8)	0.02844 (15)	0.5
C15'	0.1394 (4)	0.6163 (4)	0.9066 (3)	0.0273 (7)	0.5
H15'	0.033929	0.592377	0.877244	0.033*	0.5
C16'	0.1295 (7)	0.6379 (6)	1.0101 (5)	0.0316 (8)	0.5
H16'	0.024541	0.641524	1.056513	0.038*	0.5
C17'	0.3053 (6)	0.6519 (6)	1.0255 (4)	0.0311 (9)	0.5
H17'	0.344876	0.657661	1.092569	0.037*	0.5
C18	0.27163 (12)	0.88751 (7)	0.51432 (7)	0.01561 (14)	
C19	0.32883 (13)	0.89587 (8)	0.41174 (7)	0.01929 (16)	
H19	0.374162	0.823119	0.378549	0.023*	
C20	0.32002 (13)	1.00903 (8)	0.35821 (7)	0.02015 (16)	
H20	0.358267	1.014137	0.288616	0.024*	
C21	0.25466 (12)	1.11474 (7)	0.40751 (7)	0.01738 (15)	
C22	0.20179 (13)	1.11022 (8)	0.50943 (7)	0.01900 (16)	
H22	0.160041	1.183503	0.542488	0.023*	
C23	0.21107 (13)	0.99627 (8)	0.56233 (7)	0.01810 (15)	
H23	0.175709	0.992008	0.632360	0.022*	
N1	0.16715 (10)	0.33768 (6)	0.74663 (6)	0.01635 (13)	
H1	0.0502 (19)	0.3750 (12)	0.7331 (10)	0.020*	
O1	0.25246 (12)	0.45808 (6)	0.51977 (5)	0.02515 (15)	
O2	0.47568 (9)	0.36629 (6)	0.73864 (5)	0.01807 (12)	
OW1	0.79944 (9)	0.46463 (6)	0.69319 (5)	0.01883 (12)	
HW1A	0.697 (2)	0.4412 (14)	0.7113 (11)	0.028*	
HW1B	0.787 (2)	0.4935 (14)	0.6336 (12)	0.028*	
Cl1	0.24401 (3)	1.25584 (2)	0.33950 (2)	0.02180 (6)	

### 4'-chloro-5-oxo-N-phenyl-3-(thiophen-2-yl)-2,3,4,5-tetrahydro-[1,1'-biphenyl]-4-carboxamide monohydrate Atomic displacement parameters (Å^2^)

**Table:**

	*U*^11^	*U*^22^	*U*^33^	*U*^12^	*U*^13^	*U*^23^
C1	0.0226 (4)	0.0124 (3)	0.0183 (4)	−0.0035 (3)	−0.0030 (3)	−0.0012 (3)
C2	0.0153 (3)	0.0109 (3)	0.0178 (3)	−0.0030 (2)	−0.0001 (3)	−0.0015 (3)
C3	0.0160 (3)	0.0113 (3)	0.0170 (3)	−0.0031 (2)	−0.0003 (3)	−0.0021 (3)
C4	0.0169 (3)	0.0116 (3)	0.0181 (3)	−0.0028 (3)	−0.0004 (3)	−0.0026 (3)
C5	0.0163 (3)	0.0116 (3)	0.0186 (4)	−0.0023 (3)	−0.0021 (3)	−0.0015 (3)
C6	0.0312 (4)	0.0126 (3)	0.0169 (4)	−0.0054 (3)	−0.0030 (3)	−0.0009 (3)
C7	0.0166 (3)	0.0115 (3)	0.0156 (3)	−0.0037 (3)	0.0013 (3)	−0.0022 (2)
C8	0.0216 (4)	0.0119 (3)	0.0175 (3)	−0.0046 (3)	0.0040 (3)	−0.0021 (3)
C9	0.0250 (4)	0.0155 (4)	0.0247 (4)	−0.0027 (3)	0.0007 (3)	0.0014 (3)
C10	0.0363 (5)	0.0165 (4)	0.0250 (4)	−0.0012 (4)	0.0012 (4)	0.0026 (3)
C11	0.0457 (6)	0.0146 (4)	0.0234 (4)	−0.0086 (4)	0.0073 (4)	−0.0004 (3)
C12	0.0372 (5)	0.0194 (4)	0.0342 (5)	−0.0146 (4)	0.0059 (4)	−0.0014 (4)
C13	0.0250 (4)	0.0171 (4)	0.0301 (5)	−0.0089 (3)	0.0025 (3)	−0.0010 (3)
C14	0.0335 (5)	0.0130 (3)	0.0177 (4)	−0.0056 (3)	−0.0003 (3)	−0.0021 (3)
S1	0.0633 (7)	0.0539 (6)	0.0305 (4)	−0.0366 (6)	0.0265 (5)	−0.0197 (4)
C15	0.056 (2)	0.0290 (16)	0.0385 (16)	−0.0025 (14)	−0.0092 (16)	−0.0007 (12)
C16	0.060 (3)	0.0246 (13)	0.0310 (17)	−0.008 (2)	−0.0159 (19)	0.0009 (11)
C17	0.058 (3)	0.0328 (15)	0.0297 (16)	−0.006 (2)	0.016 (2)	0.0002 (12)
S1'	0.0355 (4)	0.0243 (3)	0.0253 (3)	−0.0025 (2)	−0.0107 (3)	−0.0027 (2)
C15'	0.0265 (13)	0.0261 (10)	0.0308 (12)	−0.0103 (12)	−0.0016 (12)	0.0012 (8)
C16'	0.041 (2)	0.0289 (16)	0.0267 (13)	−0.0130 (17)	0.0071 (15)	−0.0024 (10)
C17'	0.040 (2)	0.0309 (13)	0.0195 (11)	−0.0014 (17)	−0.0011 (14)	0.0020 (9)
C18	0.0157 (3)	0.0114 (3)	0.0200 (4)	−0.0027 (3)	−0.0031 (3)	−0.0010 (3)
C19	0.0215 (4)	0.0133 (3)	0.0230 (4)	−0.0032 (3)	0.0020 (3)	−0.0021 (3)
C20	0.0217 (4)	0.0150 (3)	0.0236 (4)	−0.0044 (3)	0.0031 (3)	0.0001 (3)
C21	0.0141 (3)	0.0119 (3)	0.0264 (4)	−0.0038 (3)	−0.0032 (3)	0.0013 (3)
C22	0.0206 (4)	0.0115 (3)	0.0252 (4)	−0.0022 (3)	−0.0037 (3)	−0.0027 (3)
C23	0.0216 (4)	0.0127 (3)	0.0204 (4)	−0.0031 (3)	−0.0037 (3)	−0.0025 (3)
N1	0.0157 (3)	0.0119 (3)	0.0216 (3)	−0.0037 (2)	0.0011 (2)	−0.0003 (2)
O1	0.0433 (4)	0.0133 (3)	0.0204 (3)	−0.0078 (3)	−0.0033 (3)	−0.0034 (2)
O2	0.0153 (3)	0.0154 (3)	0.0232 (3)	−0.0030 (2)	−0.0006 (2)	0.0004 (2)
OW1	0.0164 (3)	0.0183 (3)	0.0222 (3)	−0.0051 (2)	−0.0008 (2)	0.0001 (2)
Cl1	0.01933 (10)	0.01286 (9)	0.03293 (12)	−0.00468 (7)	−0.00022 (8)	0.00400 (7)

### 4'-chloro-5-oxo-N-phenyl-3-(thiophen-2-yl)-2,3,4,5-tetrahydro-[1,1'-biphenyl]-4-carboxamide monohydrate Geometric parameters (Å, º)

**Table:**

C1—O1	1.2324 (10)	C14—C15'	1.379 (4)
C1—C6	1.4533 (12)	C14—S1	1.7112 (15)
C1—C2	1.5233 (12)	C14—S1'	1.7126 (13)
C2—C7	1.5287 (11)	S1—C17	1.753 (7)
C2—C3	1.5396 (11)	C15—C16	1.401 (7)
C2—H2	1.0000	C15—H15	0.9500
C3—C14	1.5025 (12)	C16—C17	1.323 (6)
C3—C4	1.5362 (11)	C16—H16	0.9500
C3—H3	1.0000	C17—H17	0.9500
C4—C5	1.5005 (12)	S1'—C17'	1.755 (5)
C4—H4A	0.9900	C15'—C16'	1.404 (8)
C4—H4B	0.9900	C15'—H15'	0.9500
C5—C6	1.3511 (11)	C16'—C17'	1.321 (5)
C5—C18	1.4751 (11)	C16'—H16'	0.9500
C6—H6	0.9500	C17'—H17'	0.9500
C7—O2	1.2346 (10)	C18—C23	1.4015 (12)
C7—N1	1.3494 (10)	C18—C19	1.4022 (13)
C8—C9	1.3942 (13)	C19—C20	1.3859 (12)
C8—C13	1.3993 (13)	C19—H19	0.9500
C8—N1	1.4146 (11)	C20—C21	1.3871 (12)
C9—C10	1.3971 (13)	C20—H20	0.9500
C9—H9	0.9500	C21—C22	1.3847 (13)
C10—C11	1.3874 (16)	C21—Cl1	1.7359 (9)
C10—H10	0.9500	C22—C23	1.3896 (12)
C11—C12	1.3858 (17)	C22—H22	0.9500
C11—H11	0.9500	C23—H23	0.9500
C12—C13	1.3886 (13)	N1—H1	0.880 (13)
C12—H12	0.9500	OW1—HW1A	0.845 (15)
C13—H13	0.9500	OW1—HW1B	0.827 (16)
C14—C15	1.328 (4)		
			
O1—C1—C6	121.35 (8)	C15—C14—C3	128.6 (2)
O1—C1—C2	120.62 (8)	C15'—C14—C3	131.85 (19)
C6—C1—C2	117.94 (7)	C15—C14—S1	109.6 (2)
C1—C2—C7	109.81 (6)	C3—C14—S1	121.63 (8)
C1—C2—C3	110.89 (7)	C15'—C14—S1'	107.14 (19)
C7—C2—C3	112.27 (7)	C3—C14—S1'	120.75 (8)
C1—C2—H2	107.9	C14—S1—C17	86.2 (2)
C7—C2—H2	107.9	C14—C15—C16	122.0 (4)
C3—C2—H2	107.9	C14—C15—H15	119.0
C14—C3—C4	109.73 (7)	C16—C15—H15	119.0
C14—C3—C2	112.58 (7)	C17—C16—C15	100.9 (5)
C4—C3—C2	110.46 (7)	C17—C16—H16	129.6
C14—C3—H3	108.0	C15—C16—H16	129.6
C4—C3—H3	108.0	C16—C17—S1	120.5 (5)
C2—C3—H3	108.0	C16—C17—H17	119.7
C5—C4—C3	113.34 (7)	S1—C17—H17	119.7
C5—C4—H4A	108.9	C14—S1'—C17'	88.12 (19)
C3—C4—H4A	108.9	C14—C15'—C16'	122.5 (3)
C5—C4—H4B	108.9	C14—C15'—H15'	118.8
C3—C4—H4B	108.9	C16'—C15'—H15'	118.8
H4A—C4—H4B	107.7	C17'—C16'—C15'	101.3 (5)
C6—C5—C18	120.87 (8)	C17'—C16'—H16'	129.4
C6—C5—C4	120.24 (7)	C15'—C16'—H16'	129.4
C18—C5—C4	118.84 (7)	C16'—C17'—S1'	120.4 (5)
C5—C6—C1	123.73 (8)	C16'—C17'—H17'	119.8
C5—C6—H6	118.1	S1'—C17'—H17'	119.8
C1—C6—H6	118.1	C23—C18—C19	118.45 (8)
O2—C7—N1	124.39 (8)	C23—C18—C5	120.60 (8)
O2—C7—C2	121.41 (7)	C19—C18—C5	120.93 (7)
N1—C7—C2	114.20 (7)	C20—C19—C18	120.78 (8)
C9—C8—C13	119.43 (8)	C20—C19—H19	119.6
C9—C8—N1	124.32 (8)	C18—C19—H19	119.6
C13—C8—N1	116.20 (8)	C19—C20—C21	119.17 (9)
C8—C9—C10	119.30 (9)	C19—C20—H20	120.4
C8—C9—H9	120.3	C21—C20—H20	120.4
C10—C9—H9	120.3	C22—C21—C20	121.71 (8)
C11—C10—C9	121.32 (10)	C22—C21—Cl1	119.59 (7)
C11—C10—H10	119.3	C20—C21—Cl1	118.69 (7)
C9—C10—H10	119.3	C21—C22—C23	118.61 (8)
C12—C11—C10	119.00 (9)	C21—C22—H22	120.7
C12—C11—H11	120.5	C23—C22—H22	120.7
C10—C11—H11	120.5	C22—C23—C18	121.24 (8)
C11—C12—C13	120.61 (10)	C22—C23—H23	119.4
C11—C12—H12	119.7	C18—C23—H23	119.4
C13—C12—H12	119.7	C7—N1—C8	128.77 (7)
C12—C13—C8	120.33 (10)	C7—N1—H1	116.0 (9)
C12—C13—H13	119.8	C8—N1—H1	115.2 (9)
C8—C13—H13	119.8	HW1A—OW1—HW1B	107.1 (14)
			
O1—C1—C2—C7	28.70 (11)	C4—C3—C14—S1'	−88.62 (9)
C6—C1—C2—C7	−154.57 (8)	C2—C3—C14—S1'	147.93 (7)
O1—C1—C2—C3	153.36 (8)	C15—C14—S1—C17	−2.6 (3)
C6—C1—C2—C3	−29.90 (11)	C3—C14—S1—C17	−178.3 (3)
C1—C2—C3—C14	176.17 (7)	C3—C14—C15—C16	172.9 (4)
C7—C2—C3—C14	−60.58 (9)	S1—C14—C15—C16	−2.4 (5)
C1—C2—C3—C4	53.12 (9)	C14—C15—C16—C17	7.4 (7)
C7—C2—C3—C4	176.37 (7)	C15—C16—C17—S1	−9.5 (7)
C14—C3—C4—C5	−174.95 (7)	C14—S1—C17—C16	7.9 (6)
C2—C3—C4—C5	−50.26 (9)	C15'—C14—S1'—C17'	0.2 (3)
C3—C4—C5—C6	22.67 (11)	C3—C14—S1'—C17'	174.9 (2)
C3—C4—C5—C18	−159.71 (7)	C3—C14—C15'—C16'	−169.1 (4)
C18—C5—C6—C1	−175.02 (8)	S1'—C14—C15'—C16'	4.8 (5)
C4—C5—C6—C1	2.54 (14)	C14—C15'—C16'—C17'	−8.1 (7)
O1—C1—C6—C5	178.22 (9)	C15'—C16'—C17'—S1'	8.0 (7)
C2—C1—C6—C5	1.51 (14)	C14—S1'—C17'—C16'	−5.4 (6)
C1—C2—C7—O2	80.63 (10)	C6—C5—C18—C23	159.74 (9)
C3—C2—C7—O2	−43.22 (11)	C4—C5—C18—C23	−17.86 (12)
C1—C2—C7—N1	−99.20 (8)	C6—C5—C18—C19	−18.63 (13)
C3—C2—C7—N1	136.95 (7)	C4—C5—C18—C19	163.77 (8)
C13—C8—C9—C10	−0.70 (14)	C23—C18—C19—C20	−2.01 (13)
N1—C8—C9—C10	176.37 (9)	C5—C18—C19—C20	176.40 (8)
C8—C9—C10—C11	−0.47 (15)	C18—C19—C20—C21	0.37 (13)
C9—C10—C11—C12	1.06 (16)	C19—C20—C21—C22	1.40 (13)
C10—C11—C12—C13	−0.49 (16)	C19—C20—C21—Cl1	−179.90 (7)
C11—C12—C13—C8	−0.67 (16)	C20—C21—C22—C23	−1.44 (13)
C9—C8—C13—C12	1.27 (14)	Cl1—C21—C22—C23	179.87 (7)
N1—C8—C13—C12	−176.04 (9)	C21—C22—C23—C18	−0.28 (13)
C4—C3—C14—C15	−96.7 (3)	C19—C18—C23—C22	1.97 (13)
C2—C3—C14—C15	139.8 (3)	C5—C18—C23—C22	−176.44 (8)
C4—C3—C14—C15'	84.6 (3)	O2—C7—N1—C8	3.06 (14)
C2—C3—C14—C15'	−38.9 (3)	C2—C7—N1—C8	−177.12 (8)
C4—C3—C14—S1	77.99 (10)	C9—C8—N1—C7	8.68 (14)
C2—C3—C14—S1	−45.46 (11)	C13—C8—N1—C7	−174.16 (9)

### 4'-chloro-5-oxo-N-phenyl-3-(thiophen-2-yl)-2,3,4,5-tetrahydro-[1,1'-biphenyl]-4-carboxamide monohydrate Hydrogen-bond geometry (Å, º)

*Cg*1 is the centroid of the major component of the disordered thiophene ring (S1/C14–C17).

**Table:**

*D*—H···*A*	*D*—H	H···*A*	*D*···*A*	*D*—H···*A*
N1—H1···O*w*1^i^	0.881 (14)	1.953 (14)	2.8317 (10)	175.2 (12)
O*w*1—H*w*1*A*···O2	0.84 (2)	1.93 (2)	2.7681 (9)	171 (2)
O*w*1—H*w*1*B*···Cl1^ii^	0.83 (2)	2.81 (2)	3.0653 (7)	100 (1)
O*w*1—H*w*1*B*···O1^iii^	0.83 (2)	2.07 (2)	2.8908 (9)	172 (2)
C2—H2···S1	1.00	2.80	3.1912 (15)	104
C2—H2···O*w*1^i^	1.00	2.50	3.3269 (10)	139
C9—H9···O2	0.95	2.32	2.9124 (11)	120
C16—H16···O2^iv^	0.95	2.55	3.423 (6)	152
C17′—H17′···O2^iv^	0.95	2.58	3.511 (5)	166
C11—H11···*Cg*1^v^	0.95	2.91	3.801 (2)	158
C11—H11···*Cg*2^v^	0.95	2.86	3.757 (2)	158

Symmetry codes: (i) *x*−1, *y*, *z*; (ii) −*x*+1, −*y*+2, −*z*+1; (iii) −*x*+1, −*y*+1, −*z*+1; (iv) −*x*+1, −*y*+1, −*z*+2; (v) *x*, *y*−1, *z*.
